# An introduction to experimental phasing of macromolecules illustrated by *SHELX*; new autotracing features

**DOI:** 10.1107/S2059798317015121

**Published:** 2018-02-01

**Authors:** Isabel Usón, George M. Sheldrick

**Affiliations:** a Structural Biology, IBMB–CSIC, Baldiri Reixach 13-15, 08028 Barcelona, Spain; b ICREA, Baldiri Pg. Lluís Companys 23, 08010 Barcelona, Spain; c Department of Structural Chemistry, Georg-August Universität Göttingen, Tammannstrasse 4, 37077 Göttingen, Germany

**Keywords:** experimental phasing, MAD, SAD, direct methods, density modification, autotracing, *SHELX*

## Abstract

For the purpose of this article, experimental phasing is understood to mean the determination of macromolecular structures by exploiting small intensity differences of Friedel opposites and possibly of reflections measured at different wavelengths or for heavy-atom derivatives, without the use of specific structural models. The *SHELX* programs provide a robust and efficient route for routine structure solution by the SAD, MAD and related methods, but involve a number of simplifying assumptions that may limit their applicability in borderline cases. The substructure atoms (*i.e.* those with significant anomalous scattering) are first located by direct methods, and the experimental data are then used to estimate phase shifts that are added to the substructure phases to obtain starting phases for the native reflections. These are then improved by density modification and, if the resolution of the data and the type of structure permit, polyalanine tracing. A number of extensions to the tracing algorithm are discussed; these are designed to improve its performance at low resolution. Given native data to 2.5 Å resolution or better, a correlation coefficient greater than 25% between the structure factors calculated from such a trace and the native data is usually a good indication that the structure has been solved.

## Abstract 2

Experimental phasing of macromolecular crystals is described and explained, with the emphasis on its implementation in the programs *SHELXC*, *SHELXD* and *SHELXE*, which are also used in a number of macromolecular structure-solution pipelines.

## 1. Introduction   

Small-molecule structures are routinely solved by so-called direct methods (Usón & Sheldrick, 1999 ▸; Sheldrick *et al.*, 2011 ▸; Giacovazzo, 2014 ▸; Sheldrick, 2015 ▸), but this requires diffraction data to atomic resolution (1.2 Å or better) for typical organic structures. Occasionally, when no model is available for molecular replacement, the anomalous signal is too weak and it is not possible to soak in heavy atoms, it is also necessary to solve macromolecular structures by pure direct methods. A recent example was the solution of the parallel double-helix RNA structure of polyadenosine (Safaee *et al.*, 2013 ▸), confirming a 50-year-old prediction by Watson and Crick based on rather diffuse fibre-diffraction photographs (Rich *et al.*, 1961 ▸). For a number of years, the MAD (multi-wavelength anomalous diffraction) method (Hendrickson, 1985 ▸; Hendrickson *et al.*, 1985 ▸), exploiting small intensity differences of Friedel opposites and of reflections measured at different wavelengths, *e.g.* for selenomethionine derivatives or proteins in which elements such as iron or zinc are naturally present, was the experimental phasing method of choice. More recently, improvements in software and data quality have made it easier to solve structures by the SAD (single-wavelength anomalous diffraction) method in favourable cases using very weak anomalous scatterers, such as sulfur, that are present naturally in many proteins.

For both SAD and MAD, the positions of the substructure atoms (those with significant anomalous scattering) are first located by direct methods, which were originally developed for solving small-molecule structures (Schneider & Sheldrick, 2002 ▸). The first to use direct methods to locate substructure sites was probably Steitz (1968 ▸), and this approach was made generally applicable by Wilson (1978 ▸), who used the program *MULTAN*, at the time the direct-methods program of choice for small-molecule structures. The SAD or MAD data enable a phase shift α to be estimated that can be added to the phases calculated for the substructure to obtain starting values for the native phases. Whereas in a MAD experiment in the absence of experimental errors there would be sufficient experimental information to determine accurate phases, as shown below this is not the case for SAD. For SAD, these phases need to be improved by iterative density modification and possibly model building in order to obtain an interpretable map. Partial model building is able to extend the phase information to higher resolution. Although density modification can provide some phase improvement at low resolution, the automated chain tracing implemented in previous versions of *SHELXE* was significantly favoured by the availability of at least 2.5 Å resolution native data for SAD phasing and 3.0 Å resolution data for MAD. The chain-tracing algorithms described in this article and recently released in version 2017-1 of *SHELXE* are designed to extend these limits.

## 2. Experimental phasing procedures   

### 2.1. MAD phasing   

A MAD experiment requires data collected at at least two wavelengths. Assuming that only one type of anomalous scattering atom is present, Karle (1980 ▸) and Hendrickson *et al.* (1985 ▸) showed that the diffracted intensities can be described by
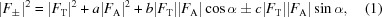
where the ‘+’ sign refers to reflection *h*, *k*, *l* and the ‘−’ sign to reflection −*h*, −*k*, −*l*. *F*_A_ is the structure factor for the substructure atoms alone, ignoring the contributions from *f*′ and *f*′′, and *F*_T_ is the total structure factor for the native macromolecule, usually including the substructure. There is one +/− pair of such equations for each wavelength, with different values for the constants *a*, *b* and *c* that can be calculated as a function of the wavelength from the complex scattering factors for the single anomalously scattering element that is assumed to be present. One of the drastic simplifications in the *SHELX* approach is that the actual values of *a*, *b* and *c* are never used! Should there be more than one anomalously scattering element present, this simply results in different peak heights for the atoms in the substructure. A beneficial side effect of these severe simplifications is that it is not necessary to know which elements are responsible for the anomalous scattering, and indeed a number of structures have been solved experimentally without this knowledge. In a MAD experiment, (1) represents an overdetermined system of equations for each reflection that can be solved to obtain values of |*F*_A_|, |*F*_T_| and α. The |*F*_A_| values may then be used to solve the substructure by direct methods using *SHELXD*. The phase shift α is then added to the calculated phases φ_A_ of the substructure to obtain starting phases φ_T_ for the full native structure,
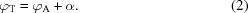



In many MAD experiments these phases will produce an interpretable map, but they can be improved further by density modification and, where appropriate, polyalanine chain tracing (see below). (2) may also be used to find φ_A_ when φ_T_ is known. The program *AnoDe* (Thorn & Sheldrick, 2011 ▸) exploits this to locate anomalous sites when the native structure is known. This provides a very useful tool for diagnosing problems in experimental phasing; in general, a peak height of at least 10σ in the resulting anomalous map (where σ is the standard deviation of the density) is required to identify substructure sites that can be found by direct methods in the substructure solution.

### 2.2. SIRAS phasing   

(1) can also be used to analyse SIRAS data in which anomalous scatterers have been introduced, for example by an iodide soak or by replacing S atoms in the native structure by Se atoms. Assuming that the native structure itself has no anomalous scatterers, there will still be three experimental measurements to determine the three unknowns |*F*_A_|, |*F*_T_| and α, so the structure can be solved by a similar approach to that used for MAD. However, SIRAS phases may be degraded by poor isomorphism.

### 2.3. SAD phasing   

In a SAD experiment there are only two experimental measurements (*F*_+_ and *F*_−_) to determine the three unknowns |*F*_A_|, |*F*_T_| and α, at first sight an impossible task. Nevertheless, this is by far the most popular experimental phasing method, partly because only one data set is required! So how does it work?

Assuming that the anomalous difference is small, subtracting (1) for *F*_−_ from (1) for *F*_+_ gives
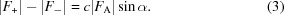



|*F*_T_| can be estimated as [|*F*_+_| + |*F*_−_|]/2. By normalizing *F*_A_ to *E*_A_, which is required anyway for substructure solution by direct methods, the constant *c* and its resolution dependence can be eliminated. However, this still leaves one equation for two unknowns, |*F*_A_| and α. The solution is to restrict the calculation to the largest (positive or negative) normalized anomalous differences, and then to assume that sinα is close to +1 (when |*F*_+_| >> |*F*_−_|) or −1 (when |*F*_+_| << |*F*_−_|). Direct methods are normally performed with only the numerically largest *E* values anyway. These approximations can be tolerated for locating the substructure atoms by direct methods because this problem is highly overdetermined: there are many more reflections than substructure sites.

Deriving starting phases for SAD is even more approximate and is also restricted to the largest normalized anomalous differences, so only a limited number of reflections can be phased reliably. To add to this problem, for reflections in centrosymmetric projections there is no Friedel difference, so these phases have a twofold ambiguity. Density modification will be required to extend the phases, and it performs best at relatively high resolution (better than 3 Å) and high solvent content. For MAD and SIRAS the resolution of the native data is much less critical, because α derived from the experimental data is then in the full range 0–360°, and reliable starting phases can be estimated for more reflections.

A detailed analysis of SAD phasing and recommendations for data preparation to obtain optimal results using it may be found in two recent papers by Terwilliger and coworkers (Terwilliger *et al.*, 2016*a* ▸,*b* ▸).

### 2.4. SIR phasing   

SIR (single-wavelength isomorphous replacement) traditionally involved soaking a protein with a heavy-metal compound to introduce ions such as mercury, platinum or lead, scaling the native and derivative data sets to each other and calculating the isomorphous differences |*F*_deriv_| − |*F*_nat_|. Applying (1) to both data sets and taking the difference gives
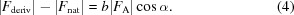



Using only the largest normalized isomorphous differences with cosα close to +1 or −1, this leads to phase shifts of 0 or 180°. As with SAD phasing, it will be necessary to improve the phases by density modification. In both cases, the initial electron-density map is a double image because the signs of the deviations from α = 90 or 270° (SAD) or 0 or 180° (SIR) have not been determined. However, there is a subtle but important difference: for SAD one image is positive and the other is negative, so an iterative density-modification procedure that sets negative density to zero will improve the map, but for SIR both images are positive, so density modification will be less effective. Since the isomorphous derivative will also have an anomalous signal that can be enhanced further on a beamline with a tuneable wavelength, SIRAS should usually be used in preference to SIR. Alternatively, the SIR phase ambiguity can also be resolved by using multiple heavy-atom derivatives (MIR or MIRAS). Although MIR is primarily of historical interest, it can still be useful for very large structures at low resolution.

### 2.5. RIP phasing   

Instead of adding a heavy atom, the isomorphous derivative can be produced by radiation damage, for example during a synchrotron X-ray data collection or by selectively breaking disulfide bonds with a UV laser (Nanao *et al.*, 2005 ▸). The analysis is then analogous to SIR. Although the isomorphism is generally better than with heavy-atom soaks, scaling the data sets to each other can still be critical, and density modification suffers from the same disadvantages as SIR.

## 3. Substructure solution   

### 3.1. Data truncation   

**Figure 1:**
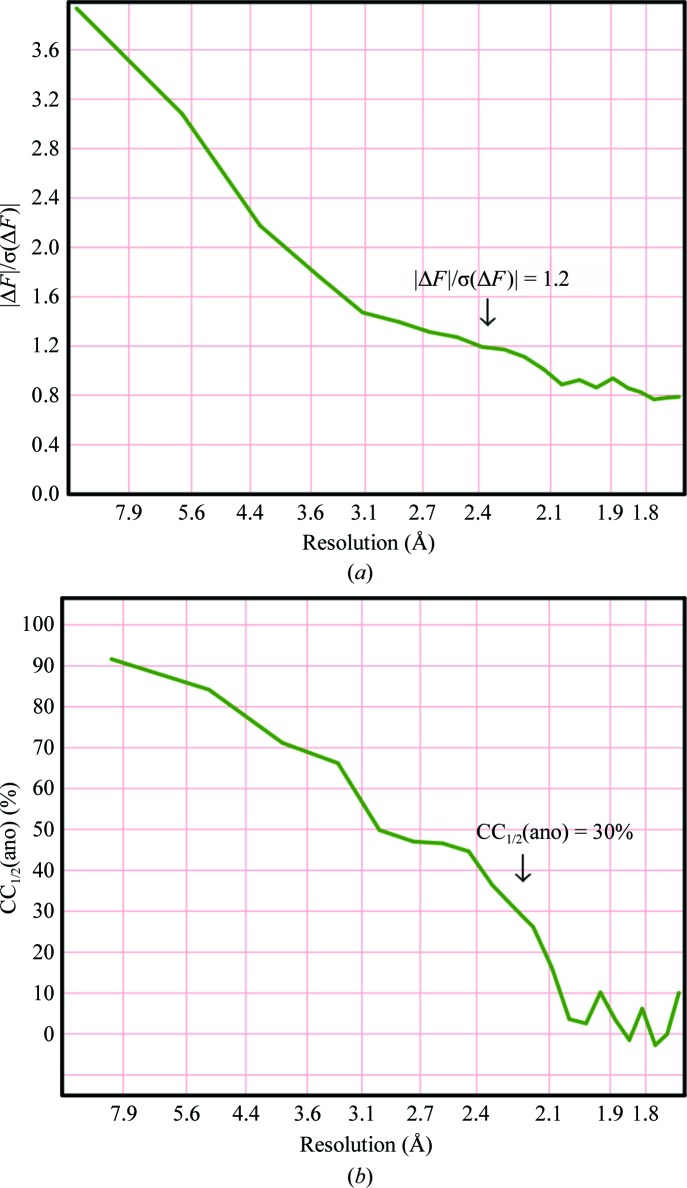
Resolution dependence of (*a*) mean 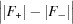 divided by its standard deviation and (*b*) CC_1/2_(ano) for viscotoxin A1 (Pal *et al.*, 2008 ▸).

The direct methods used to determine the substructure require normalized structure factors (*E* values), which has the effect of weighting up the high-resolution data. It may then be necessary to truncate the resolution of the data used to obtain the anomalous differences to improve their signal-to-noise ratio. Typically, it will be necessary to truncate these data to a resolution about 0.5 Å lower than the diffraction limit, and indeed this is the default in some automated pipelines (for example *HKL*2*MAP*; Pape & Schneider, 2004 ▸). Two criteria that are frequently used to decide where to truncate the data are the ratio of the anomalous difference to its standard deviation (Fig. 1 ▸ *a*) and the correlation coefficient CC_1/2_(ano) (Karplus & Diederichs, 2012 ▸) between two random subsets of the anomalous difference data (Fig. 1 ▸ *b*). The ratio of the anomalous difference to its standard deviation requires an accurate estimate of the standard deviation, but a good test of this is whether this ratio asymptotes to the theoretical value of (2/π)^1/2^ = 0.798 for pure noise at high resolution. CC_1/2_(ano) is independent of the standard deviation but requires unmerged data. Substructure location based on maximum-likelihood targets (Read & McCoy, 2018 ▸) does not require the data to be truncated because the maximum-likelihood function weights the data appropriately.

### 3.2. Special action for disulfide groups   

When the anomalous signal does not extend to sufficient resolution to resolve disulfides, it has been standard practice to treat them as ‘super-S atoms’. At low resolution (worse than about 3 Å) this is probably justified, and at resolutions higher than about 2 Å the individual S atoms can usually be resolved. A feature of the intermediate resolution range that appears to be unique to *SHELXD* is the ability to search for disulfides with a fixed S–S distance in the peak-search routine of the dual-space direct methods. This results in more accurate phases. *SHELXD* also provides facilities for the location of magic triangles (a sticky ligand containing an equilateral triangle of three I atoms; Beck *et al.*, 2008 ▸).

### 3.3. Critical parameters for *SHELXD* and fine-tuning the substructure solution   

*SHELXD* requires an approximate estimate of the number of substructure sites; this should, if possible, be within about 20% of the true value. In the case of a heavy-atom soak this can be difficult to estimate and some trial and error may be required. Experience suggests that the number of requested sites should be chosen so that the weakest site has an occupancy of about 0.2 relative to 1.0 for the top site. If NCS is present, it may also give an indication as to which of the weaker sites are correct. For large substructures it may be necessary to use a large number of random trials; in at least one example, one million trials were required to find one correct solution (fortunately *SHELXD* is highly parallel).

The procedure described here is simple and robust, but involves several severe simplifications. In borderline cases it may be worth using the LLG (log-likelihood gain) to refine the substructure solutions, for example using the programs *SHARP* (La Fortelle & Bricogne, 1997 ▸; Bricogne *et al.*, 2003 ▸), *CRANK*2 (Skubák & Pannu, 2013 ▸) or *Phaser* (Read & McCoy, 2011 ▸; Bunkóczi *et al.*, 2015 ▸).

## 4. Density modification   

### 4.1. General principles   

Although a high-quality MAD data set can produce an immediately recognizable map, for SAD phasing it is essential to improve the map by density modification. Density modification attempts to make the density look more like the density expected for a macromolecule. If we performed an inverse Fourier transform of the unmodified density we would recover the initial phases, so we need to make a chemically sensible modification to the density before inverting it back. For example, the density determined by X-ray diffraction should never be negative, so simply setting all negative density to zero should be a good start and should help to remove the false-negative image that arises from the twofold ambiguity in SAD phasing. This immediately explains why density modification works better at high native resolution and at high solvent content, because there is then less accidental cancellation of the positive and negative images. However, for electron diffraction the correct image is partly positive (from the positively charged nuclei) and partly negative (from the electrons), so density modification would be much less effective.

### 4.2. The sphere-of-influence algorithm   

Classical density modification, as pioneered by B.-C. Wang, divides the map into protein and solvent regions, and flattens the solvent density (Wang, 1985 ▸). Usually, the protein regions are assumed to be those with the largest density fluctuations. *SHELXE* avoids the necessity of locating and smoothing the boundary between protein and solvent by using the sphere-of-influence algorithm (Sheldrick, 2002 ▸), which is possibly unique to *SHELXE*. In this algorithm, the variance *V* of the density is calculated for a spherical surface of radius 2.42 Å (a typical 1,3 distance in a macromolecule) around each voxel in the map. For voxels with a low variance the density at the voxel is ‘flipped’ (ρ′ = −γρ, where γ may be set by the user but is typically 1.1). Voxels with a high variance are essentially left unchanged, but a sharpening function may be applied similar to that used by Foadi *et al.* (2000 ▸). The *SHELXE* procedure is related to the γ-correction of Abrahams (1997 ▸), except that the γ-correction requires an explicit solvent boundary. For intermediate values of the variance, *SHELXE* applies a weighted mean of the corrections for the protein and solvent regions.

### 4.3. Enantiomorph discrimination   

Direct methods are blind to the hand of the substructure. After correcting the density, the program calculates the contrast and connectivity of the map for both hands. The contrast is the variance of *V* over all voxels in the map and the connectivity measures the fraction of contiguous voxels that have high electron densities. High values of the contrast are desirable, and if one hand has an appreciably higher contrast then it is the correct substructure. It is also possible that both hands are correct, *i.e.* when the substructure is centrosymmetric, for example when there are only two heavy-atom sites with similar occupancies in a monoclinic space group.

### 4.4. The free-lunch algorithm   

*SHELXE* has an option to extend the resolution of the data used for generating electron-density maps (Usón *et al.*, 2007 ▸). For relatively high-resolution data (2 Å or better) and especially if the solvent content is high (greater than 50%) this can produce spectacular results. Polyalanine chain tracing is also effective under these conditions but is slower and limited to polypeptides, whereas the free-lunch data extrapolation can be used for other macromolecules such as polynucleotides. By default this approach is also used to phase and estimate the intensities of missing low-resolution reflections.

## 5. Model building   

The generation of a polyalanine trace in *SHELXE* is designed to improve the phases but does not include identification of the individual amino-acid residues and matching the sequence as performed in *ARP*/*wARP* (Lamzin *et al.*, 1999 ▸), *RESOLVE* (Terwilliger, 2003 ▸) and *Buccaneer* (Cowtan, 2006 ▸).

If secondary structure is apparent in a noisy map, combining the phase information derived from a partial trace with σ_A_ weights (Read, 1986 ▸) is an obvious way of improving it. The stereochemical constraints implicit in the model are very effective in extending phases to higher resolution, where the experimental phase information is weaker or not present at the onset. Conversely, introducing wrongly traced stretches rapidly leads to deterioration of the map. The *SHELXE* main-chain tracing is conservative and gradual, occasionally rendering an interpretable map from phases characterized by mean phase errors (MPE) above 75°, as shown in §[Sec sec6]6, especially when the solvent content is high.

### 5.1. Polyalanine chain tracing   

Polyalanine chain tracing (Sheldrick, 2010 ▸) can only be used for proteins and, unlike density modification, it is sensitive to the resolution. It also increases the program runtime considerably; however, a parallel version is under development. The first, and currently rate-limiting, step is a search for template fragments to seed the autotracing. Randomly positioned starting fragments are refined by a simplex algorithm to optimize a weighted sum of the modified densities at atomic positions. The weights correspond to the atomic numbers, but negative weights are used for selected points that should not be occupied by atoms. The highest ranking fragments are extended N- and C-terminally by fitting peptide units to the density, again using a simplex algorithm in which the main-chain φ and ψ torsion angles are varied, taking ‘no-go regions’ into account. These are regions that are too close to existing atoms or symmetry elements. After the first tracing cycle, stored polypeptides from the previous tracing cycle are also employed as seeds for the tracing. If noncrystallographic symmetry operators have been extracted from the positions of the substructure atoms, they are applied to the traced chains to generate further potential starting fragments. Finally, traces are combined by splicing them together. The program also tries to bridge gaps in the trace where the density is consistent with this.

### 5.2. Additional constraints in chain tracing   

**Figure 2:**
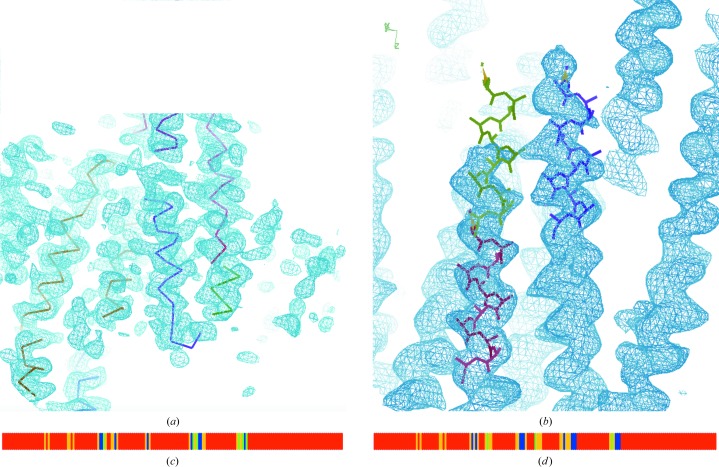
Issues in map tracing and additional constraints. (*a*) The trace corresponding to an electron-density map of the coiled coil autophagy-related protein 38 at 2.4 Å resolution illustrates how the helical geometry is degraded in the regions where the map is poor. (*b*) shows how an originally placed seed (lime) continuing a correct seed (red) is refined by the unrestrained simplex into the density of a neighbouring helix (purple). The neighbouring helix happens to be in the reversed direction. (*c*) Seed coverage in the first autotracing cycle of fibronectin for the unrestrained *versus* (*d*) restrained simplex refinement of β-sheets. In (*c*) and (*d*) the sequence numbers increase from left to right and the r.m.s.d. of the seeds *versus* the correct structure is represented in blue (<0.36 Å), green (0.5 Å) and yellow (<1 Å). Restraining the simplex refinement of the seeds (*d*) renders a better and more accurate coverage than in (*c*).

**Figure 3:**
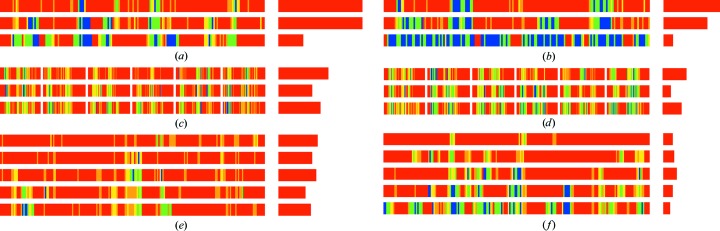
Three examples of tracing evolution: (*a*, *b*) apoferritin, (*c*, *d*) autophagy-related protein 38 and (*e*, *f*) titin A168-A169. (*a*), (*c*) and (*e*) show standard polyalanine tracing, (*b*) shows restrained helical tracing with 14-residue seeds, (*d*) with 12-residue helical seeds extended by sliding and (*f*) with two-stranded antiparallel β seeds. The full sequence is shown on the left, with blue for C^α^ atoms within 0.3 Å, green for those at 0.6–0.3 Å, yellow for those at 1.0–0.6 Å, orange for those at 2.0–1.0 Å and red for no match. Incorrectly traced residues are shown on the same scale on the right.

The procedure described by Sheldrick (2010 ▸) starting from template α-helices and common tripeptides has been refined and extended to make it more suitable for lower resolution and for initial maps with large phase errors. A more constrained, albeit slower, procedure is required, especially in the case of structures where the secondary structure involves predominantly β-strands. Even for helical stretches, the electron density may present gaps and poor correlation with the atomic model, especially in bent regions, as shown in Fig. 2 ▸(*a*). Therefore, additional templates have been incorporated into *SHELXE*. Longer helices of up to 14 residues may now be used as starting seeds. Whatever the length, the simplex is a powerful minimizer for pulling a solution from a local into a global minimum, so the random seeds tend to refine to a small number of over-represented positions (Fig. 2 ▸ *b*). To increase the coverage, the search fragments are tethered weakly to their starting positions while allowing free rotation. At lower resolution it is also desirable to restrain the extension of the longer helical fragments in the initial autotracing cycles so that the sum of the ψ angle of one residue and φ of the next residue remains close to −105°. This restraint is not applied in the final autotracing cycle, by which time the phases are better, so that it does not hinder chain extension beyond the end of the helix into connecting loops or turns. Fig. 3 ▸ shows a comparison of the previous and the current algorithm, illustrating the evolution of the polypeptide trace in the course of three global cycles in the case of the helical protein apoferritin. The main improvement in the constrained algorithm appears to be a reduction in the number of incorrect traces, which would otherwise cause the tracing to deteriorate in successive cycles.

For particularly long helices, such as those present in coiled coils and at resolutions below 2 Å (Caballero *et al.*, 2018 ▸), an alternative, more constrained helical extension is worth trying (Figs. 2 ▸ *b* and 3 ▸ *b*). A helical template is translated to extend the polypeptide chain, a restrained refinement is then performed to fit the electron-density map, and it is then broken up into tripeptides that are individually refined. The process is iterated to extend the N- and C-terminal ends of the initial template until the density correlation decreases or extension would collide with a previously traced region.

Outside the more constant helical regions, accurate geometrical representation in the seeds requires a broader choice of fragments and limiting their size. The three most common tripeptides (one helical and two β-strands; Pavelcik & Pavelcikova, 2007 ▸) are used as before to seed phasing. As β-strands are known to be more constant in the way they build sheets than in their backbone geometry, the original tripeptide fragments have now been extended to include a parallel as well as an antiparallel two-stranded template, where each strand is a tripeptide. Both templates have been derived from β-sheets in the PDB through geometrical clustering (Sammito *et al.*, 2013 ▸). The tracing can be parameterized to locate the more frequent antiparallel strands, parallel strands or both. As in the case of the helical seeds, a random search is followed by a tethered positional optimization. The two strands in the template are then decoupled and extended independently at both ends.

### 5.3. Criteria for accepting chains   

The following criteria are combined into a single figure of merit to decide whether to accept the traced chains.

(i) The fit to the density should be good.
(ii) The chain must be long enough (in general at least seven amino acids). Longer chains are given a higher weight.
(iii) Most of the φ, ψ angle pairs should be in well populated regions of the Ramachandran plot, taking into account that some glycines may be present.
(iv) There should be significant density at a point extrapolated in the N→H direction to 2.9 Å from the N atom. This is intended to detect hydrogen bonds.
(v) There should be a well defined secondary structure for most of the trace. Thus, the φ, ψ angle pairs should be similar for consecutive amino acids.

### 5.4. Preliminary refinement of the trace   

When no more chains can be extended, a *B* value (isotropic temperature factor) is refined for each substructure atom (if they are present in the native structure) and for each traced amino-acid residue. If the structure is large, adjacent residues are combined and given the same *B* value to reduce the number of parameters. The hope is that if an incorrect residue is present, its *B* value will refine to a large value, effectively smearing it out. The atom coordinates are not refined. After this refinement is complete, the program calculates a correlation coefficient CC for the structure against the native data. This turns out to be a rather good indication as to whether the structure is correct; for a resolution of 2.5 Å or better, a CC value of 25% or higher makes it likely that the structure is solved. However, in the presence of translational NCS false positives with high CC values are not uncommon (Caballero *et al.*, 2018 ▸), but the correct solution has a higher CC. The phases calculated from the trace are combined with the experimental phases to calculate the map to be traced in the next global cycle (if any). σ_A_ weights are used for the phase combination.

Table 1 ▸ summarizes the results obtained with *SHELXC*/*D*/*E* for the SAD phasing and main-chain tracing of 21 structures with resolutions ranging from 1.2 to 3.0 Å.

### 5.5. Starting from molecular-replacement fragments   

An alternative use of *SHELXE* is to trace from fragments that have been found by molecular replacement (Thorn & Sheldrick, 2013 ▸). This forms a key stage in the programs *ARCIMBOLDO* (Millán *et al.*, 2015 ▸) and *AMPLE* (Bibby *et al.*, 2012 ▸). In general, this requires higher resolution data (typically better than 2.5 Å) than building a trace from experimental phases (typically better than 3 Å).

## 6. Tracing tests   

**Figure 4:**
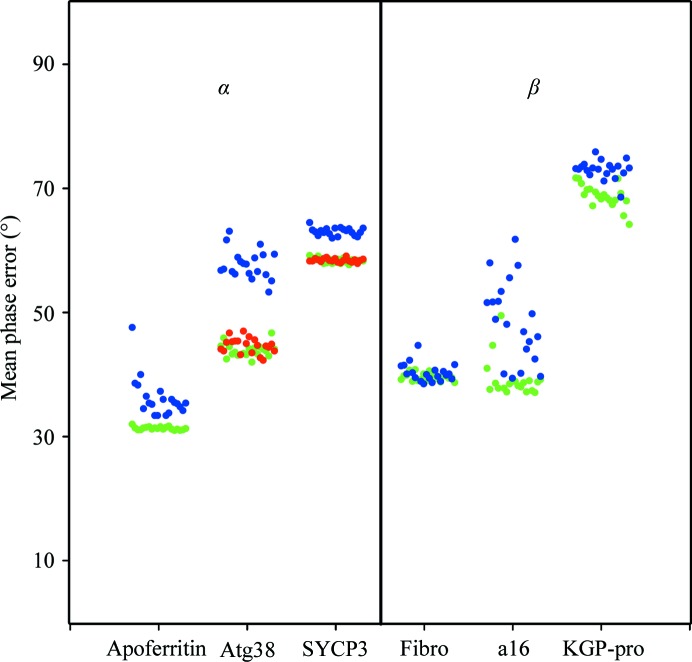
Phasing results for 20 runs comparing standard (blue) *versus* restrained tracing from new seeds (long, tethered helices or two-stranded β seeds in green; sliding of helical seeds in red) in the case of apoferritin with 14-­residue helical seeds, autophagy-related protein 38 (atg38) with helix sliding to extend the 12-residue helical seeds, synaptonemal complex SYCP3 with both types of helical tracing, fibronectin (fibro) with two-stranded β seeds, titin protein A168-A169 (a16) with two-stranded β-­sheets and Kgp prodomain (KGP-pro).

**Figure 5:**
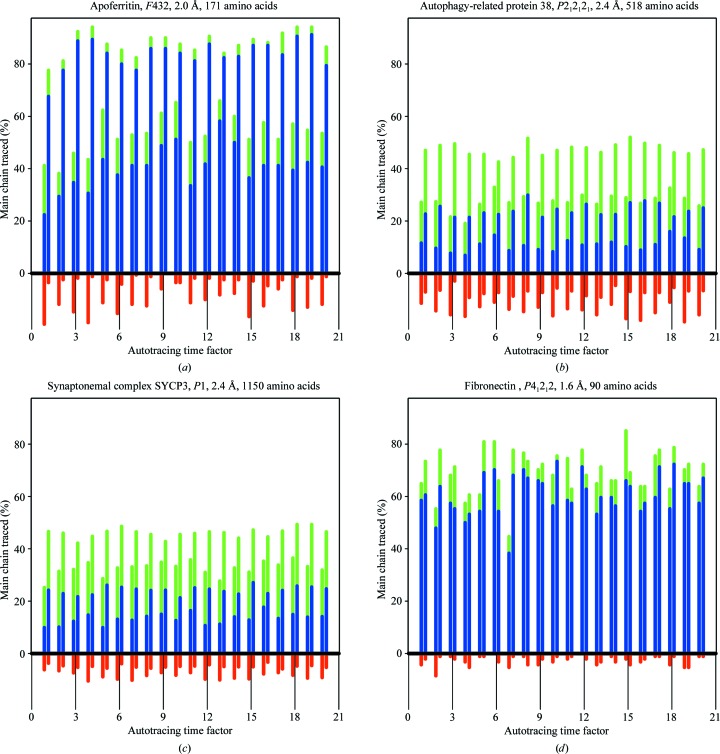

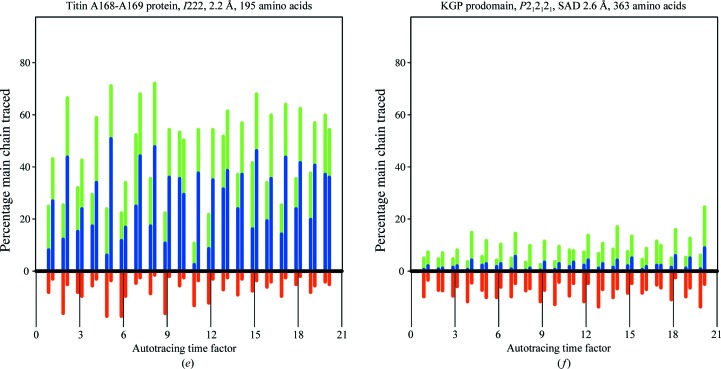
Percentage of the main chain traced correctly within 1 Å r.m.s.d. (green), within 0.5 Å r.m.s.d. (blue) or incorrectly traced (red) for (*a*) apoferritin with 14-­residue helical seeds, (*b*) autophagy-related protein 38 with helix sliding to extend the 12-residue helical seeds, (*c*) synaptonemal complex SYCP3 with both types of ­helical tracing, (*d*) fibronectin with two-stranded β seeds, (*e*) titin protein A168-A169 with two-stranded β-sheets and (*f*) Kgp prodomain.

Six structures (Table 2 ▸) have been used to test and illustrate the new features in *SHELXE* as described above. SAD data for apoferritin and titin protein A168-A169 are from Mueller-Dieckmann *et al.* (2007 ▸) and those for fibronectin are from Rudiño-Piñera *et al.* (2007 ▸); Kgp prodomain (Pomowski *et al.*, 2017 ▸) is a difficult all-β protein with a low solvent content. The C-terminal domain of autophagy-related protein 38 (atg38; Ohashi *et al.*, 2016 ▸) and human synaptonemal complex protein 3 (SYCP3; Syrjanen *et al.*, 2014 ▸) are large coiled-coil proteins, which for the purpose of this study were phased from SAD data at resolutions of 2.4 Å even when originally solved by MAD or SIRAS. The anomalous scatterer substructures summarized in Table 2 ▸ were located with *SHELXD*. As the autotracing algorithm starts from random seeds entailing an inherent variability, for each of these structures 20 *SHELXE* jobs were run, varying the time parameter (-t) to obtain 20 different sets of seeds. The results are plotted in Fig. 4 ▸, displaying the weighted mean phase error (MPE) characterizing the final electron-density map obtained for each of the six proteins with the various tracing algorithms and otherwise equivalent parameterization. Fig. 5 ▸ shows the main-chain coverage of the trace.

Apoferritin is a helical structure for which data to a resolution of 2.0 Å from a cadmium-containing crystal were available. Helices make up more than 80% of the structure, with 25-amino-acid long helices, so longer helical seeds of 14 alanines were used and tethered to their original positions in the simplex refinements, and extension of the seeds explored only helical torsion angles in the first two cycles. Fig. 4 ▸ shows the MPE obtained with three cycles of standard (blue) and restrained (green) autotracing. The best case for both alternatives renders comparable values (Table 1 ▸), decreasing the initial value of 73° to 31° (restrained) and 33° (standard), but the results of the restrained procedure are more even, as comparable values are obtained in all 20 runs incorporating positional tethering restraints and helical extension, whereas not all of the unrestrained runs converged. Looking at Fig. 3 ▸, which displays the evolution of the traces obtained in the best-scored trace for both algorithms, a higher percentage of initially incorrectly traced polypeptide is apparent in the unrestrained case. Correspondingly, in the final tracing cycle a more complete and correct trace is obtained in the restrained case. This pattern is general within the two sets of apoferritin runs, as illustrated in Fig. 5 ▸. The restrained runs on the right display a more complete and correct trace coverage of the true structure than the unrestrained runs on the left.

Atg38 and SYCP3 are coiled coils of 511 and 1150 amino acids, respectively, phased in this study using only one of the available data sets by selenium and iodine SAD at a resolution of 2.4 Å. Three autotracing cycles were run for the former and five for the latter. For the long helices in coiled coils, extending helical seeds by translation is appropriate to bridge the occasional regions where the map is less clear and tracing would be interrupted or degraded. This issue is illustrated in Fig. 2 ▸(*a*), which displays a standard trace of atg38, where the geometry of the traces for regions that should be helical becomes degraded by the poor electron density in the noisy map. Fig. 4 ▸ shows that in both cases restrained autotracing leads to a lower MPE indicative of a more accurate trace with higher coverage, as is also apparent from Fig. 5 ▸.

Three β-sheet structures, fibronectin, titin protein A168-A169 and the prodomain of Kgp, were also tested. Fibronectin at a resolution of 1.6 Å renders comparable maps and traces with both algorithms, except for one outlier, thus dismissing the concern that the restrained autotracing would handicap tracing at high resolution. Restraints are released in the final cycle, so that other than the increase in computing time there should be no drawback for the new algorithm. The titin protein A168-A169 at 2.2 Å resolution profits from the restrained refinement and, as illustrated for a pair of equivalently parameterized runs for the alternative algorithms, restraining reduces the number of incorrect traces. This generally enables a more correct chain to be built (Fig. 5 ▸), leading to a lower MPE (Fig. 4 ▸). The Kgp prodomain at 2.6 Å resolution is a borderline case that nevertheless illustrates the advantage of the restrained tracing.

## 7. Conclusions   

This paper provides an overview of experimental phasing using *SHELXC*, *SHELXD* and *SHELXE*, concentrating on SAD phasing, which is currently the most popular form of experimental phasing. It describes various improvements in the algorithms that can make the difference between success and failure in borderline cases. A number of innovations have been added to *SHELXE* in the main-chain tracing and are designed to improve the performance at lower resolution and for all-β structures. Evaluation of the accuracy of the polypeptide traced after each cycle shows that the main improvement of the constrained algorithm is a reduction in the number of false traces rather than an immediate increase in the number of correct traces. This builds up in the following cycles as the map improves, whereas if there are too many poor traces in the initial cycles it may not be possible to recover. In general, a CC of 25% or higher for the main-chain trace against native data to 2.5 Å resolution or better indicates a successful solution.

## Figures and Tables

**Table 1:** Summary of density-modification and tracing statistics for test structures CC(HA) is the substructure CC for the heavy atoms against the anomalous data from *SHELXE*, *n* is the first tracing-cycle number with CC > 30%, CC is the best CC within three cycles of cycle *n*, %CA/1 Å is the percentage of C^α^ atoms within 1 Å of their correct positions and wMPE is the weighted mean phase error for this trace. The -z option was not used (since it is not very reliable). The *SHELXE* parameters given are -s, solvent fraction; -m, number of density-modification cycles; -h, number of heavy-atom sites to use. -a for autotracing was used in all cases.

Structure and source of data	*SHELXE* parameters	Space group	*d* (Å)	CC(HA)	*n*	α/β	CC (%)	%CA/1 Å	wMPE (°)
Apoferritin†	-s0.61 -h8	*F*432	2.00	29.0	4	77/7	47.6	91.2	29.8
hARH3†	-s0.44 -h16	*P*2_1_2_1_2_1_	1.82	13.2	4	67/6	39.2	81.9	34.9
Insulin†	-s0.44 -h6	*I*2_1_3	1.80	30.8	1	47/20	56.5	96.1	23.3
Lysozyme pH 4.5†	-s0.39 -h16	*P*4_3_2_1_2	1.84	40.6	1	44/13	36.7	69.8	33.9
Lysozyme pH 8.0†	-s0.36 -h13	*P*4_3_2_1_2	1.84	26.6	2	43/13	41.7	82.2	35.2
NBR1 PB1†	-s0.62 -h3	*P*6_3_22	2.15	15.3	2	30/33	50.6	88.4	38.2
PPE-Ca†	-s0.41 -h11	*P*2_1_2_1_2_1_	1.84	29.7	1	15/44	45.2	92.1	36.4
PPE-Na†	-s0.40 -h13	*P*2_1_2_1_2_1_	2.15	41.1	3	15/43	35.4	70.1	42.2
Proteinase K†	-s0.39 -h13	*P*4_3_2_1_2	1.95	34.3	5	32/26	34.3	68.5	42.8
Thaumatin†	-s0.57 -h15	*P*4_1_2_1_2	2.00	24.2	2	13/44	39.2	81.2	29.a
Thermolysin†	-s0.46 -h10	*P*6_1_22	1.98	26.7	1	42/17	42.3	84.9	33.6
Trypsin *P*3_1_21†	-s0.38 -h16	*P*3_1_21	1.82	35.5	1	14/40	41.8	82.1	34.3
Trypsin‡	-s0.46 -h17	*P*2_1_2_1_2_1_	1.20	19.9	1	11/36	41.2	85.3	28.0
Elastase‡	-s0.40 -h12	*P*2_1_2_1_2_1_	1.37	15.9	1	15/43	41.5	96.2	31.5
Viscotoxin A1§	-s0.53 -h12	*P*4_3_2_1_2	1.70	23.5	1	45/22	52.4	100.0	24.1
Viscotoxin A3¶	-s0.32 -h14	*P*2_1_2_1_2	2.20	28.3	4	45/19	37.6	65.2	44.4
Titin A168-A169†	-s0.67 -h4 -m20	*I*222	2.20	12.4	15	5/64	34.9	70.6	36.2
SYCP3††	-s0.58 -h14	*P*1	2.41	13.1	—	96/1	23.6	28.8	62.3
Autophagy-related protein 38‡‡	-s0.70 -h10	*P*2_1_2_1_2	2.44	7.3	19	87/0	32.5	47.2	43.7
Fibronectin§§	-s0.37 -h9	*P*4_1_2_1_2	1.60	19.8	11	4/54	39.3	34.0	48.6
MG491¶¶	-s0.50 -h4	*P*4_1_2_1_2	3.00	42.9	21	79/4	34.6	21.1	63.4

† Using data from Mueller-Dieckmann *et al.* (2007 ▸).

‡ Using data from Debreczeni, Bunkóczi *et al.* (2003 ▸).

§ Using data from Pal *et al.* (2008 ▸).

¶ Using data from Debreczeni, Girmann *et al.* (2003 ▸).

†† Using data from Syrjanen *et al.* (2014 ▸).

‡‡ Using data from Ohashi *et al.* (2016 ▸).

§§ Using data from Rudiño-Piñera *et al.* (2007 ▸).

¶¶ Using data from Martinelli *et al.* (2015 ▸).

**Table 2:** Summary of density modification and autotracing with *SHELXE* for six test structures The *SHELXE* parameter line applies to the case for which results are summarized: -m, number of density-modification cycles; -h, number of heavy-atom sites to use; -s, solvent fraction; -a, autotracing; -q, length of helix template; -Q, use helical extension in tracing; -B1, use antiparallel β templates for tracing.

					Standard tracing	Restrained tracing
ID	Space group	*N*_res_	CASE	*d* (Å)	wMPE (°)/NTRACE/CC (%)	wMPE (°)/NTRACE/CC (%)
Apoferritin	*F*432	171	SAD, 8 Cd	2.0	-m20 -h8 -s0.61 -a3 -q -t13	-m20 -h8 -s0.61 -a3 -q14 -t18
					33.4/123/33.0 (8.2)	31.0/160/50.4 (1.2)
Autophagy-related protein 38	*P*2_1_2_1_2	518	SAD, 10 Se	2.5	-m10 -h10 -q -s0.7 -a3 -t18	-m10 -h10 -Q -s0.7 -a3 -t16
					53.3/275/24.6 (11.0)	42.3/327/32.3 (7.3)
						-m10 -h10 -q14 -s0.7 -a3 -t12
						42.0/311/33.6 (5.8)
Human synaptonemal complex protein 3	*P*1	1150	SAD, 15 I	2.4	-m15 -h15 -s0.6 -q -a5 -t9	-m15 -h15 -s0.6 -a5 -Q -t4
					62.0/613/25.4 (7.3)	55.5/775/34.6 (2.4)
						-m15 -h15 -s0.6 -a5 -q14 -t17
						56.3/762/33.5 (2.5)
Terminal organelle protein MG491	*P*2_1_2_1_2	556	SAD, 4 Se	3.0	-m10 -h4 -s0.5 -q -a5 -t20	-m10 -h4 -s0.5 -a5 -Q -t19
					65.6/272/33.4 (12.2)	56.4/299/39.5 (4.8)
						-m10 -h4 -s0.5 -a5 -q14 -t14
						58.9/282/38.0 (5.2)
Fibronectin	*P*4_1_2_1_2	90	SAD, 9 S	1.6	-m20 -h9 -s0.38 -a5 -t9	-m20 -h9 -s0.38 -a5 -B1 -t20
					38.5/68/34.8 (4.3)	38.7/63/35.4 (1.1)
Titin protein A168-A169	*I*222	195	SAD, 4 S	2.2	-m20 -h4 -s0.67 -a5 -t10	-m20 -h4 -s0.67 -a5 -B1 -t18
					39.4/124/28.9 (5.6)	37.1/132/30.6 (2.0)
Kgp prodomain	*P*2_1_2_1_2_1_	254	SAD, 5 I	2.6	-m10 -h -s0.5 -v0.5 -a20 -t17	-m10 -h -s0.5 -v0.5 -a20 -B1 -t20
					68.6/107/17.6 (5.3)	64.2/115/20.4 (5.0)
